# Banging the drum: evolutionary and cultural origins of music and its implications for psychiatry

**DOI:** 10.1192/bjb.2023.44

**Published:** 2023-10

**Authors:** Gerry Rafferty, Gurjot Brar, Mara Petrut, David Meagher, Henry O'Connell, Paul St John-Smith

**Affiliations:** 1 University of Limerick, Limerick, Ireland; 2 Retired consultant psychiatrist

**Keywords:** Transcultural psychiatry, complementary therapies, individual psychotherapy, group psychotherapy, psychosocial interventions

## Abstract

There is growing interest in music-based therapies for mental/behavioural disorders. We begin by reviewing the evolutionary and cultural origins of music, proceeding then to discuss the principles of evolutionary psychiatry, itself a growing a field, and how it may apply to music. Finally we offer some implications for the role of music and music-based therapies in clinical practice.

The number of studies on music-based therapies for mental/behavioural disorders published yearly has increased steadily over the years, accompanied by growing interest in the therapeutic utility of music. Recently, its use has been researched in various medical conditions^1–3^ and applied in physiological,^4^ behavioural^5–7^ and psychological therapies.^8,9^ This panoply of uses reflects an evolutionary underpinning of music and its role in integrating complex physiological, emotional and cognitive functions in the human brain.

Music may be described as the art of producing pleasing or expressive combinations of audible tones with pitches and sequences to produce melody, rhythm and harmony. It may utilise vocal and/or instrumental sounds combined in such a way as to produce beauty of form and expression of emotion. Every known human culture appears to engage in some form of music. Therefore, it may be treated as a universal cultural phenomenon worthy of evolutionary investigation.

The evolutionary origins of music are highly contentious. Some argue that choral or vocal forms of music are associated with the origin of language, which itself remains a highly debated issue. Although non-verbal forms of rhythmic sounds are seen in many other organisms, there is much disagreement surrounding what is considered music and what function non-mating calls have. The origins of music and its evolutionary aspects are still debated, and it is unclear to what extent they can be understood from a Darwinian perspective. However, this does not axiomatically exclude us from considering music through an evolutionary lens. Such examination can still be scientifically fruitful, even if it remains incomplete.

## A brief history of music

There is a reasonable consensus that some form of music first arose in the Palaeolithic period. Most Palaeolithic instruments have been found in Europe and date back to the Upper Palaeolithic. It is quite probable that singing emerged far before this time, although this cannot currently be scientifically confirmed owing to the Eurocentric bias in archaeological evidence.

Possibly the oldest discovered musical instrument is a flute from the Divje Babe cave in Slovenia, dated between 43 000 and 82 000 years ago. It was made from a young cave bear femur and is thought to have been used by Neanderthals. If true, it suggests that Neanderthals and Homo sapiens may have acquired musical abilities from a common ancestor before they split roughly half a million years ago. This suggests that the use of non-verbal music is even more ancient than posited in theories that focus on the ‘development of language’ and artifacts. It is likely that earlier, simpler instruments existed before the flute, similar to objects that are common in later hunter-gatherer societies, such as rattles, shakers and drums. The lack of other instruments from and before this time may be due to their use of biodegradable materials, such as reeds, gourds, skins and bark.^10^ For instance, a painting in the Cave of the Trois Frères in France, dating to around 15 000 bce, is thought to depict a shaman playing a musical bow.^11^

Animals use organised sounds to convey warnings which are generated below the conscious level. Higher mammals such as whales and dolphins communicate by meaningful complex sounds, although the content of what is conveyed and whether it is purely interindividual remains a subject of debate. Dolphins appear to communicate with each other on an individual basis, during both work and play, but this behaviour is suggested to be innate rather than purposefully cognitive.^12^ There is evidence that higher primates use tools to make sounds and beats to organise social behaviour. This is believed to be the evolutionary jump that led to organised, deliberate sound production with cognitive control of meaning.^13^ It may also be the point at which tonal archetypes first appeared in human evolution, and these seem to persist in our collective human awareness to this day.

## Current theories

There appear to be three main avenues for considering the origin of music in relation to language: (a) beginning as a proto-language as a result of adaptation leading to language as we know it; (b) a ‘spandrel’ (a phenotypic by-product of evolution) occurring as a result of language development; or (c) music and language share a common antecedent.

Darwin speculated that music may have arisen as part of an elaborate form of a sexual selection process perhaps primarily arising from mating calls. This is perhaps the first relevant scientific/biological theory on the origin of music. It first appeared in Darwin's 1871 book *The Descent of Man, and Selection in Relation to Sex*.^14^ His ideas have been criticised as there is no controlled evidence that either human sex is ‘more musical’ and thus no evidence of sexual dimorphism within that line of inquiry. However, despite the formal sexual selection hypothesis being biologically unlikely, even the most casual observer could hardly fail to notice that love, sex and other romantic content are major themes in Western music.

That music arose alongside language, both of which might descend from a ‘shared precursor’, was considered first by the 19th-century biologist Herbert Spencer, who was an important early proponent of this theory. In the 21st century, several scholars have supported this view, particularly the archaeologist Steven Mithen.^15^

Further theories of the evolutionary function of music expound on selection pressures based on practical needs such as: assisting in organising cohesive labour, improving ease and range of long-distance communication, enhancing communication with the divine or supernatural, assisting in social cohesion among families and tribes and as a means of frightening predators or human enemies. According to Nettl,^16^ music may have had two origins, ‘from speech (logogenic) and from emotional expression (pathogenic)’. This theory was first proposed by the musicologist Curt Sachs. Reflecting on the diversity of music around the world, Sachs noted that some music confines to either a communicative or expressionistic form, suggesting that these aspects developed separately. It is the latter that mostly interests psychiatrists.

## The evolutionary perspective

What are the evolutionary and cultural origins of music? Does an understanding of music and its evolution have implications for our understanding of emotions and mental disorders? Can the power of music be harnessed to enhance psychotherapeutic interventions?

Evolutionary psychiatry seeks to blend the theory of evolution with current understanding of mental disorders. It has primordial roots in ethology and biology, furthered by evolutionary psychology and medicine, which are gaining much traction in recent times.^17^ Knowledge of the ‘proximate’ neuropsychological effects of music, combined with a deeper understanding of its ‘ultimate’ or phylogenetic evolutionary underpinnings, can lead to the development of therapies that help deliver person-centred approaches to treating specific disorders (Table 1). For example, individual playlists have potential for use in addressing emotion dysregulation and self-destructive cognitions seen in emerging emotionally unstable personality disorder (EUPD) in younger people.^19^

**Table 1:** The evolutionary approach to music and causation based on Tinbergen's four questions^18^

Proximate	Ultimate
Causation/mechanism	Function/adaptation
Identification of the perceptual stimuli and psychological, social and material processes involved in music	Describes the possible evolutionary advantage(s) conferred by music itself
Development/ontogeny	Evolution/phylogeny
Describes how the music has influenced early experience and how it is modified over the lifespan of an individual	Describes the evolution of music over time in a species

Consider the drum and beats riffs that persist from primitive tribal societies to complex techno music of today. The archetypal or *a priori* nature of certain rhythms seems to be identifiable across cultures and geography: for example, the traditional Irish ‘Marcshlua Uí Néill’ (‘O'Neill's March’) and the Māori haka share the same basic beat and musical structure. The composition of Native American music for rituals has clear parallels with the structure of Gregorian chants. The neurobiology of these transcultural music constructs would seem to be coherent. Functional positron emission tomography (PET) scanning shows^20^ that the same parts of the limbic system, in particular the amygdala, the hippocampus and the temporal limbic system, seem to be activated by all iterations of music from these various cultures.

Primates achieve bonding groups of up to 50 through social grooming, which takes up about 20% of their day, via the endorphin systems.^21,22^ Opioids and endorphins create feelings of warmth, relaxation and trust, facilitating social bonding. This system is conserved in humans and underpinned by the same mechanisms.^23^ In human groups exceeding 50, it becomes necessary to ‘groom at a distance’ for practical reasons. Over time, this appears to have led to the development of a suite of behaviours, including singing, dancing, feasting, laughter, rituals of religion and emotional storytelling.^24^ Many of these have been shown to trigger the endorphin system: for example, the wall-sit test revealed an increase in pain thresholds after watching emotionally arousing drama,^25^ and using naltrexone as an antagonist to block endorphin uptake revealed similarly increased thresholds after engaging in group synchronised dance;^26^ naltrexone blocking of mu-opiods reduced social bonding in ritual contexts;^27^ and positron emission tomography (PET) showed increases in endogenous opioid release when engaging in social laughter and eating enjoyable food.^28,29^

Music appears to have played a large role in rituals and facilitating group bonding/cohesion to maintain bonds within large communities. Indeed ancestrally, the threat of exile appears to have been a prominent motivator to remain in groups.^30^ The incredibly poor physical and mental health outcomes of modern loneliness are well documented.^31,32^ A significant predictor of mental and physical health, well-being and longevity is the number and quality of close friendships,^33–36^ which is not unique to humans, appearing in other mammals.^37–39^

There exists a substantial body of evidence on the ‘proximate’, i.e. direct, neurobiological effects of music on mental processes. The anxiolytic, euphoriant and subliminal impacts on mood and well-being have been reported extensively.^40^ There is clear evidence for the euphoriant effect of listening to music, an effect that can be attenuated with agents such as naloxone.^41^

Music is deployed to affirm complex emotional states. Nearly all cultures use music to set the emotional cadence of inherent and vital rituals. Both celebration and grief have universally recognisable musical ‘soundtracks’. It is widely accepted that music can effectively convey happiness, sadness, love or anger. Music can promote tribal identity or a sense of belonging, independent of the lyric, if one indeed exists. Consider the haka, the bagpipes or the tonal evocations of Native Americans.^42,43^ The Jungian perspective raises the concept of archetypal forces at play, in keeping with the evolutionary perspective outlined above. Therefore, the study of the evolution of music provides a potentially unifying theory that incorporates multiple domains, from neurobiology to psychoanalysis.

Considering the development of what we would describe as music, the introduction of lyrics also represents an important step. Rather than collective or universally understood tonal patterns conveying emotions, the use of language conveys cognitive and therefore neocortical function. The words of national anthems or love songs convey far greater meaning or power than the music alone. Music has evolved from sounds with meaning, such as warning or tribal identity, to become opera or the lyrics of Bob Dylan.

## Conclusions

By analysing the phylogenetic origins of music, we can see a clear progression from simple forms and uses to a more complex communication hierarchy. Therefore, it can be inferred that exposure to music is likely to provoke deeply emotional and cognitive responses, and this opens the way for the development of individualised therapeutic uses of music in a variety of psychiatric and emotional disorders. If our understanding of music has evolved with us then it seems logical to conclude that music can be the medium by which people of diverse backgrounds and different phenomenological experiences can connect in a meaningful and therapeutic way.

The general and mental health benefits of participating in making music, being in a band, playing an instrument or being part of a choir are well documented. There is less known about the targeted use of music either as a therapeutic intervention or as a way of expressing oneself by recognising difficult-to-articulate cognitions and emotions. There is still less evidence on helping patients describe their symptomology by referencing music that resonates with them. Emerging evidence shows clear opportunities for the use of music-based therapy in treating younger people with emerging EUPD, and these treatment principles can be widely generalised to other disorders and age groups.

The importance of music to the individual was recognised by Darwin himself: ‘If I had my life over again, I would have made a rule to read some poetry and listen to some music at least once every week’.^44^

## About the authors

**Gerry Rafferty**, Senior Registrar, Department of Psychiatry, University of Limerick, Limerick, Ireland; **Gurjot Brar**, Senior Registrar, Department of Psychiatry, University of Limerick, Limerick, Ireland; **Mara Petrut**, Registrar, Department of Psychiatry, University of Limerick, Limerick, Ireland; **David Meagher**, Chair of Psychiatry and Consultant Psychiatry, Department of Psychiatry, University of Limerick, Limerick, Ireland; **Henry O'Connell**, Associate Clinical Professor, University of Limerick, Limerick, Ireland; and Consultant Psychiatrist, Laois–Offaly Mental Health Service, Portlaoise, Ireland; **Paul St John-Smith**, retired consultant psychiatrist; independent scholar; and Chair of the Evolutionary Psychiatry Special Interest Group, Royal College of Psychiatrists, London, UK.

## Data Availability

Data availability is not applicable to this article as no new data were created or analysed in this study.
